# Genome Sequencing and Comparative Analysis of Three *Hanseniaspora uvarum* Indigenous Wine Strains Reveal Remarkable Biotechnological Potential

**DOI:** 10.3389/fmicb.2019.03133

**Published:** 2020-01-21

**Authors:** Nicoletta Guaragnella, Matteo Chiara, Angela Capece, Patrizia Romano, Rocchina Pietrafesa, Gabriella Siesto, Caterina Manzari, Graziano Pesole

**Affiliations:** ^1^Institute of Biomembranes, Bioenergetics and Molecular Biotechnologies, CNR, Bari, Italy; ^2^Department of Biosciences, Biotechnology and Biopharmaceutics, University of Bari “A. Moro”, Bari, Italy; ^3^Dipartimento di Bioscienze, Università degli Studi di Milano, Milan, Italy; ^4^Scuola di Scienze Agrarie, Forestali, Alimentari ed Ambientali, University of Basilicata, Potenza, Italy

**Keywords:** non-*Saccharomyces* yeasts, *Hanseniaspora uvarum*, genome sequencing and annotation, *Hanseniaspora* species, comparative genomics, flocculation

## Abstract

A current trend in winemaking has highlighted the beneficial contribution of non-*Saccharomyces* yeasts to wine quality. *Hanseniaspora uvarum* is one of the more represented non-*Saccharomyces* species onto grape berries and plays a critical role in influencing the wine sensory profile, in terms of complexity and organoleptic richness. In this work, we analyzed a group of *H. uvarum* indigenous wine strains as for genetic as for technological traits, such as resistance to SO_2_ and β-glucosidase activity. Three strains were selected for genome sequencing, assembly and comparative genomic analyses at species and genus level. *Hanseniaspora* genomes appeared compact and contained a moderate number of genes, while rarefaction analyses suggested an open accessory genome, reflecting a rather incomplete representation of the *Hanseniaspora* gene pool in the currently available genomes. The analyses of patterns of functional annotation in the three indigenous *H. uvarum* strains showed distinct enrichment for several PFAM protein domains. In particular, for certain traits, such as flocculation related protein domains, the genetic prediction correlated well with relative flocculation phenotypes at lab-scale. This feature, together with the enrichment for oligo-peptide transport and lipid and amino acid metabolism domains, reveals a promising potential of these indigenous strains to be applied in fermentation processes and modulation of wine flavor and aroma. This study also contributes to increasing the catalog of publicly available genomes from *H. uvarum* strains isolated from natural grape samples and provides a good roadmap for unraveling the biodiversity and the biotechnological potential of these non-*Saccharomyces* yeasts.

## Introduction

Species of the genus *Hanseniaspora*, widely known as “apiculate yeasts” due to their lemon-shaped cell morphology, are largely distributed in different environments and includes many species. Expressly, the species *Hanseniaspora uvarum* is frequently found on mature fruits and particularly on grapes (Zott et al., 2008; Wang et al., 2015a). *H. uvarum* is also frequently isolated from other fermented beverages, such as cider, palm wine and cashew juice, tequila, sugar-cane aguardente (Owuama and Saunders, 1990; Lachance, 1995; Morais et al., 1997; Valles et al., 2007). This yeast was isolated also from exotic substrates, such as African coffee and in chocolate production (Masoud et al., 2004; Illeghems et al., 2012; Batista et al., 2016).

*Hanseniaspora uvarum* shows antagonistic properties against the development of molds responsible for fruit spoilage and it was proposed as biocontrol agent against plant pathogens, such as *Botrytis cinerea* on grapes and strawberries and *Penicillium* spp. on citrus (Long et al., 2005; Cai et al., 2015). On the other hand, *H. uvarum* is considered a spoilage yeast in some processes, such as yogurt, orange juice, beer and honey production (Wiles, 1950; Kosse et al., 1997; Renard et al., 2008; Pulvirenti et al., 2009).

In nature, *H. uvarum* was isolated from different sources, such as soils, plants, insects, birds, mollusk and shrimps, moreover it was occasionally found as clinical isolate in humans where it is considered as opportunistic (Albertin et al., 2016). The widespread diffusion and economic importance of this yeast species demonstrate the high potentiality for application of *H. uvarum* in food biotechnology and especially in the wine sector in terms of product and process innovation (Tristezza et al., 2016; Capozzi et al., 2019; Berbegal et al., 2017).

In winemaking, these yeasts constitute more than 50% of the total yeast population (Fleet and Heard, 1993), but due to their sensitivity to increasing ethanol concentration, they are gradually replaced by *Saccharomyces cerevisiae*, the principal wine yeast (Fleet, 2003; Capece et al., 2005). Further studies demonstrated also the existence of interactions mechanisms between *H. uvarum* and *S. cerevisiae* during alcoholic fermentation. *S. cerevisiae* can produce killer toxin (Schmitt and Neuhausen, 1994), and release yet unidentified antimicrobial peptides, which play an important role in reducing non-*Saccharomyces* yeast population (Albergaria et al., 2010; Wang et al., 2015b).

In the past, this yeast species was traditionally considered as undesirable in winemaking and the addition of sulfites was the traditional way to prevent the risk of its growth at the beginning of the vinification process besides other approaches to limit its proliferation during fermentation (Comitini and Ciani, 2010). The main limit of this species in winemaking is the production of high levels of acetic acid and ethyl acetate, although some authors showed that not all the strains formed high levels of volatile acidity and many of them produced similar levels to those of *S. cerevisiae* (Bezerra-Bussoli et al., 2013).

However, considering that in the first days of fermentation *H. uvarum* reaches a very high cell density, it is expected that this yeast contributes significantly to fermentation affecting wine characteristics also if *S. cerevisiae* is added as starter culture (Capece and Romano, 2019). The actual trend in winemaking has re-evaluated the role of non-*Saccharomyces* yeasts due to their potential beneficial properties that contribute to increasing the sensory complexity of wines (Berbegal et al., 2017). Indigenous strains of *H. uvarum* are associated to specific terroir, produce fruity esters and possess a high enzymatic activities (esterases, β-glucosidases, lipases, and proteases), which might contribute to increase the sensory wine complexity (Belda et al., 2016; Tofalo et al., 2016a). In particular, strains of this species have been reported to exhibit β-glucosidase activity 6.6-fold higher than that of indigenous *S. cerevisiae* strains. This characteristic can be correlated with the increase of volatile compounds contents, such as free terpene, volatile phenols and C13-norisoprenoid, possessing a sensory impact (Martin et al., 2018). Selected *H. uvarum* strains were used as mixed starter cultures, both as co-inoculation and sequential inoculation with *S. cerevisiae*, to increase the wine organoleptic quality during industrial production, although until now no *H. uvarum* strain is commercialized as oenological starter culture (Tristezza et al., 2016; Petruzzi et al., 2017; Roudil et al., 2019). Moreover, *H. uvarum* has been shown to be compatible with *S. cerevisiae* and *O. oeni* in a simultaneous inoculation for the industrial production of regional typical wines, further supporting the use of mixed starter formulation as a promising approach in industrial application (Capozzi et al., 2019). In addition, the presence of *H. uvarum* has been detected during organic must fermentation in selected wine grape growing regions, supporting the significance to preserve biodiversity of these non-*Saccharomyces* native yeasts (Suzzi et al., 2012).

Despite this obvious importance of *H. uvarum* in wine fermentation, data on its genetic makeup is quite scarce. Karyotyping approaches suggested the presence of 7 to 9 chromosomes, with high variability between different isolates (Esteve-Zarzoso et al., 2001; Cadez et al., 2002), whereas the mitochondrial genome of *H. uvarum* has an exceptional structure among fungi, as it is represented by a short, linear DNA molecule (Pramateftaki et al., 2006). Recently, some brief reports on whole-genome approaches have appeared for *Hanseniaspora* strains, underlining the growing interest in this yeast, but with limited information regarding genome annotations (Giorello et al., 2014; Sternes et al., 2016; Seixas et al., 2017).

In this study, 26 *H. uvarum* strains isolated from spontaneous fermentation of grapes from different origin or source were subjected to a preliminary screening for genetic and phenotypic variability. Three strains, possessing different genetic and phenotypic traits, were selected and submitted to genome sequencing and assembly. Comparative genomics analysis at the genus and species levels was performed to identify candidate genes of potential biotechnological relevance.

## Materials and Methods

### Yeast Strains and Growth Conditions

*Hanseniaspora uvarum* strains used in this study are listed in Table 1. All strains have been isolated during spontaneous grape must fermentations, performed at lab-scale from grapes of different varieties and directly collected in the vineyard. All the strains were grown on YPD medium (1% yeast extract; 2% peptone; 2% glucose; 2% agar) and maintained at 4°C.

**TABLE 1:** List of *Hanseniaspora uvarum* strains used in this work.

Strain	Source	Code	References
H318	Cannonau (Sardinia)	H1	
20EI5	Aglianico (Basilicata)	H2	Capece et al., 2005
20EII5	Aglianico (Basilicata)	H3	
F12	Bosco (Liguria)	H4	
CBS6617	Banana (Japan)	H5	CBS-KNAW Fungal Biodiversity Centre
RA7-4	Inzolia (Sicily)	H6	Capece et al., 2011
H319	Cannonau (Sardinia)	H7	
H320	Cannonau (Sardinia)	H8	
3EII1	Aglianico (Basilicata)	H9	Capece et al., 2005
7EI3	Aglianico (Basilicata)	H10	
7EII4	Aglianico (Basilicata)	H11	
10EII4	Aglianico (Basilicata)	H12	
18EII1	Aglianico (Basilicata)	H13	
10EII2K20	Aglianico (Basilicata)	H14	
5EII3	Aglianico (Basilicata)	H15	
4EIII5	Aglianico (Basilicata)	H16	
20EI2	Aglianico (Basilicata)	H17	
13EII5	Aglianico (Basilicata)	H18	
11EIII5	Aglianico (Basilicata)	H19	
15EII4	Aglianico (Basilicata)	H20	
CBS5074	Apple must (Chile)	H21	CBS-KNAW Fungal Biodiversity Centre
CBS8130	Muscat grape (Russia)	H22	
CBS2589	Grape must (Italy)	H23	
CBS2587	Fruit must (Austria)	H24	
CBS5934	Cider (Illinois, United States)	H25	
DBVPG 6718	Muscatel grape (Crimea, Russia)	H26	Industrial Yeasts Collection DBVPG

### Genomic DNA Isolation and Genotypic Characterization

Genomic DNA was isolated by using a synthetic resin (Instagene Bio-Rad Matrix) as previously described in Capece et al. (2011). Genotypic characterization was performed, as previously described for the characterization of non-*Saccharomyces* strains (Cadez et al., 2002; Capece et al., 2005; Andrade et al., 2006). The M13 (Cadez et al., 2002), P80 (Capece et al., 2005) and microsatellite-primed PCR (MSP-PCR) by using the synthetic oligonucleotide (GTG)_5_ and (GACA)_4_, were used for RAPD-PCR and MSP-PCR, respectively. The repeatability of these techniques was assessed in two independent amplification reactions with three repetitions, using *H. uvarum* reference strain DBVPG 6718. Amplification reactions were performed in a final volume of 50 μL containing 10 μL of Taq Polymerase 5X Buffer (Promega), 4.0 μL of 25 mM MgCl_2_ (Promega, Milan, Italy), 1 μL of 10 mM dNTP (Promega), 5 μL of 5 μM primer, 0.25 μL (5 U/μL) of Taq DNA polymerase (Promega) and 5 μL of template, with sterile water, added up to final volume. The thermal cycler was programed as follows: initial denaturation at 95°C for 5 min, 35 cycles at 94°C for 1 min for denaturing, 1 min at the primer-specific annealing temperature [54°C for M13 and P80, 52°C for (GTG)_5_ and 43°C for (GACA)_4_], 2 min at 72°C for extension and a final step at 72°C for 5 min. PCR products were analyzed by electrophoresis in 1.2% agarose gel, prepared in 1X TAE buffer (40 mM Tris–Acetate, 1 mM EDTA, pH 8.0). The gels were run at 100 V for 90 min, stained with SYBR^®^ Safe (Invitrogen, United States) and captured by the Gel Doc^TM^ XR + system (Bio-Rad). The profiles were analyzed with FQWest software v.4.5 (Bio-Rad), using Pearson correlation and the dendrogram was constructed using UPGMA (tolerance 1%, optimization 0.5%). The cophenetic correlation was used to ascertain the consistency of the obtained cluster.

### Technological Characterization

For technological characterization, both extracellular β-glucosidase activity and resistance to sulfur dioxide (SO_2_) have been determined. Quantitative screening of the extracellular β-glucosidase activity was performed following the protocol described by Mendes Ferreira et al. (2001). Strains were inoculated in 20 mL of YPD liquid medium and, after 48 h of incubation at 26°C, 1 × 10^6^ cells/mL were inoculated in a liquid medium composed by yeast nitrogen base without aminoacids (0.67%), glucose (2%) and 0.4 mL of ferric ammonium citrate solution (1% w/v). Flasks were incubated in an orbital shaker at 150 rpm and 26°C for 24 h. Extracellular β-glucosidase activity was determined in 1 ml of supernatant, recovered by centrifugation at 3000 rpm for 10 min. Enzymatic activity was evaluated by determining the amount of *p*-nitrophenol (pNP) released from the *p*-nitrophenyl-β-D-glycoside (pNPG) by adding 0.2 mL of pNPG solution (5 mmol/L) in citrate-phosphate buffer (citric acid 0.1 M, Na_2_HPO_4_ 0.2 M, pH 5) to 0.2 mL of each supernatant fluid and incubating at 30°C for 1 h. The reaction was stopped by adding 1.2 mL of Na_2_CO_3_ solution (0.2M). The amount of pNP released was determined spectrophotometrically at 400 nm. The enzymatic activity was quantified using a standard curve of pNP ranging between 10 and 150 nmol/mL. Results were expressed as nmol of pNP released for mL for hours.

Resistance to SO_2_ was tested by evaluating strain fermentative performance in natural red grape must (pH 3.4; sugars 225 g L^–1^; yeast assimilable nitrogen (YAN) 234 mg L^–1^) pasteurized at 100°C for 20 min, supplemented with 50 mg/L of total SO_2_ (added as potassium metabisulfite), as reported in Capece et al. (2011). Pasteurized grape must without SO_2_ addition was used as a control. The fermentations were performed at 26°C. Each sample was inoculated with 10^7^ cell/mL from pre-cultures grown for 24 h in 5 mL of YPD liquid medium. The samples with SO_2_ were inoculated after 30 min of SO_2_ addition.

The SO_2_-resistance was expressed as the ratio between fermentative vigor (amount of CO_2_ produced at the third day of fermentation) of strains in the presence of SO_2_ and without SO_2_.

### Genome Sequencing, *de novo* Assembly and Annotation

DNA library preparation was performed by the means of TruSeq DNA Nano Sample Prep kit (Illumina, San Diego, CA, United States), according to manufacturer’s instruction. Inserts size ranges were approximately between 200 and 500 bp. The library obtained from the H2 strain was sequenced on the Illumina NextSeq500 platform. A total of 30 M of 100 bp paired-end reads were produced. While libraries obtained from the H4 and H20 strains were sequenced on an Illumina MiSeq platform in order to obtain 2 × 200 bp paired-end reads. Reads were subjected to quality trimming using the Trimmomatic program, with default parameters (Bolger et al., 2014). Quality trimmed reads were subsequently subjected to assembly Spades (Prjibelski et al., 2014) using the default parameters and the following range of kmers (33,55,77,99) for the 100 bp reads and (33,55,77,99,121) for the 200 bp reads. Scaffolding was performed, using SSPACE (Boetzer and Pirovano, 2014). Gene annotation was performed using the Augustus program (Stanke et al., 2006), with gene models derived from *S. cerevisiae* and using the default cut-off value of 0.4 for the posterior probability.

### Clusters of Orthologous Genes (COGS)

All against all BlastP (Altschul et al., 1990) were performed using the BLOSUM80 matrix and accepting only best reciprocal hits with an *e*-value ≤ 1e-5, which covered at least 40% of the protein length, and where “second-best” hits produce bit scores < 90% of that associated with the best match. Putative COGs were established as groups of best reciprocal blast hits, using a custom utility available at https://github.com/cvulpispaper/compute_aai_and_cogs.

### Rarefaction Analyses of Core and Accessory Genomes

For each number of organisms considered (*H. uvarum* strains and *Hanseniaspora* species, respectively) the inferred sizes of core and accessory genomes were recorded for all the possible combinations of genomes. To avoid possible ascertainment biases in the comparison at genus level only one representative genome assembly was considered for species where more than one assembly was available. For *H. uvarum* and for *H. vineae* the genome assembly of the AWRI3580 and T02/19AF strain were considered, respectively. Plots were prepared showing mean and standard deviation of these statistics.

### Identification of Heterozygous Sites and Calculation of Genomic Identity Levels

Where available (see Supplementary Table S1), heterozygous sites were inferred directly from the reference genomic assemblies based on IUPAC ambiguity codes. Alternatively, raw sequencing reads were obtained from public sequence repositories and aligned to their respective reference genome assembly in order to identify heterozygous sites (see Supplementary Table S1). Alignments were performed using the Bowtie2 (Langmead and Salzberg, 2012) software with default parameters, variant calling was performed by the means of the Freebayes program, again using default parameters (Garrison and Marth, 2012).

All heterozygous sites were masked using a custom Perl script. Complete genome assemblies were aligned using the Minimap2 program (Li, 2018) using the asm20 preset. Genomic identity levels were estimated directly from the Minimap2 output files by the means of a custom Perl script, available at https://github.com/matteo14c/minimap2_to_genome_identity.

Unfortunately due to lack of data (sequencing reads not deposited in any publicly available database) masking of heterozygous sites was not possible for the *H. uvarum* strain 34-9, *H. uvarum* strain CBA6001, *H. vineae strain* T02/19AF and *H. vineae strain* T02/05AF as indicated in Supplementary Table S1. Considerations regarding the relatively low levels of heterozygosity (from 0.18 to 0.81% depending on the species. Supplementary Table S1), if compared to the average level of identity between species (average 76.45%), would, however, suggest that this is not likely to have a considerable impact on the final clustering.

### Hierarchical Clustering of Genomes

Hierarchical clustering was performed using the R (version 3.4.4 2018-03-15) implementation of the Neighbor-Joining algorithm from the cluster package (Maechler et al., 2019).

### Protein Domain Enrichment Analyses

PFAM protein domains were annotated to predict protein-coding genes with the pfam_scan.pl program, using both the Pfam-A and Pfam-B domain models from the Pfam32.0 release of the Pfam database, with default parameters (Finn et al., 2016). The number of occurrences of each Pfam domain in each genome was counted using a Perl custom script. A simple R (version 3.4.4) script based on the hypergeometric distribution and implementing a Bonferroni correction was used to compute the *p-*value for the over-representation of the domains in each group.

### Statistical Treatment of Data

Statistical analyses were performed using the Stats package as provided by the R programing language (version 3.4.4) R Core Team (2018). R: A language and environment for statistical computing. R Foundation for Statistical Computing, Vienna, Austria URL https://www.r-project.org/.

### Flocculation Test

Flocculation test was performed according to the method described by Suzzi and Romano (1991), with small changes. Flocculation capacity was tested in liquid YNB (yeast nitrogen base), inoculating 24-h cells. Flocculation capacity was assessed by eye after 2, 15, and 20 days of incubation at 26°C. The flocculation levels, with small changes. Flocculation capacity was tested in liquid YNB (yeast nitrogen base), inoculating 24-h cells. Flocculation capacity was assessed by eye after 2, 15, and 20 days of incubation at 26°C. Flocculation levels were classified based on a rating scale from 0 (not flocculent) to 5 (very flocculent).

## Results

### Genotypic and Technological Characterization of *H. uvarum* Strains

All the *H. uvarum* indigenous strains used in this work and their relative geographical origin are reported in Table 1.

**FIGURE 1:**
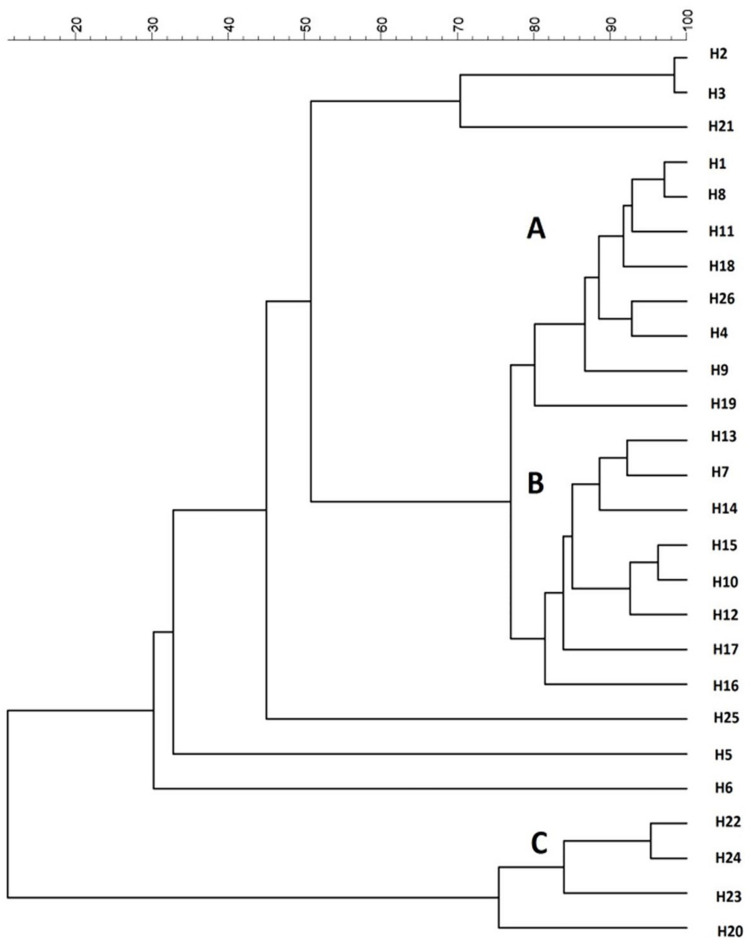
Cluster analysis with UPGMA method and Pearson distance of 26 *H. uvarum* profiles based on MSP-PCR with (GTG)_5_ primer.

All the 26 strains were subjected to genotypic and phenotypic characterization. *H. uvarum* strain typing was performed by using four different PCR methods based on RAPD analysis and MSP-PCR as described in Materials and Methods. According to our results, the (GTG)_5_ primer showed the best discrimination between the *H. uvarum* strains considered in this work, whereas other primers generated profiles that were too similar and/or had low levels of reproducibility (data not shown). Molecular profiles obtained by PCR fingerprinting with the (GTG)_5_ primer were clustered using the UPGMA algorithm with Pearson correlation-based distance measures, three major groups (A, B, C) were identified based on a similarity cut-off. Six isolates that did not show similarity with any other isolate were considered singletons (Figure 1). No particular correlation between the source of isolation of the strains and genotypic clustering was observed, with the notable exception of group B, which is composed mainly (7/8) by strains isolated from the Aglianico grape variety.

Phenotypic characterization was performed by measuring two fermentative parameters, such as the levels of β-glucosidase activity and the resistance to SO_2_ during the early stages of fermentation (Table 2).

**TABLE 2:** Technological characterization of *H. uvarum* strains.

*S*trains	β-glucosidase activity^∗^	FVR^∗∗^
H1	94.5 ± 4.7	1.00
H2	137 ± 3.4	1.00
H3	nd	1.00
H4	87 ± 5.9	0.75
H5	62 ± 2.9	1.00
H6	54.5 ± 6.1	0.89
H7	99.5 ± 2.8	1.00
H8	27 ± 1.9	1.00
H9	34.5 ± 2.4	0.95
H10	74.5 ± 2.1	1.00
H11	42 ± 4.5	0.78
H12	22 ± 8.5	0.77
H13	37 ± 4.3	1.00
H14	132 ± 2.6	0.70
H15	22 ± 4.9	0.62
H16	22 ± 5.1	1.00
H17	77 ± 2.6	0.50
H18	62 ± 1.6	0.79
H19	102 ± 3.2	1.00
H20	47 ± 1.8	1.00
H21	12 ± 0.4	1.00
H22	132 ± 4.8	1.00
H23	52 ± 1.3	1.00
H24	17 ± 1.8	0.52
H25	77 ± 5.8	0.79
H26	75 ± 6.1	1.00

*^∗^β-glucosidase activity expressed as nmol of pNP released for mL/h. ^∗∗^FVR = SO_2_ resistance expressed as the ratio between the fermentative vigor in presence of 50 mg/L of SO_2_ (FV_*with SO*_2_) and without SO_2_ addition (FV_*without SO*_*2*_). nd, not determined.*

Quantitative screenings for extracellular β-glucosidase activity revealed high levels of variability among the strains. Of note, only the H3 strain did not exhibit detectable levels of β-glucosidase activity. In two strains (H21 and H24), a weak enzymatic activity was observed (12 and 17 nmol pNP ml^–1^ h^–1^), whereas four strains (H2, H14, H19 and H22) exhibited a high level of activity (higher than 100 nmol pNP ml^–1^ h^–1^). From a biotechnological point of view, high levels of β-glucosidase activity have been previously associated with increased hydrolysis of bound monoterpenes, which can enhance the fruity character of the wines (Rodriguez et al., 2004; Jolly et al., 2014).

The strains were also tested for the influence of SO_2_ on the fermentative activity as this compound is normally added to crushed grapes and *H. uvarum* species is known to be highly sensitive. The resistance of the strains to SO_2_ was assessed by measuring the fermentative vigor in the presence of 50 mg/L of SO_2_ (FV_with SO__2_). For each strain, the ratio (FVR) between FV with SO_2_ and the fermentative vigor without SO_2_ addition (FV without SO_2_) was used to express the SO_2_ resistance level. Values of this ratio similar to 1 show no differences between strain fermentative vigor with or without sulfur dioxide addition, whereas values lower than 1 indicate that the SO_2_ addition determines a reduction of fermentative vigor of the strains, indicating strains very sensitive to this compound. As reported in Table 2, fifteen strains were not affected by SO_2_ (FVR values equals to 1), whereas the remaining strains showed varied levels of sensitivity. The H24 strain, in particular, displayed a marked reduction of about 50% of the fermentative vigor (FVR values of 0.52). On the basis of these results, three wild *H. uvarum* strains, H2, H4 and H20, which were assigned to different groups according to (GTG)_5_ profiles (Figure 1) and exhibited different levels of resistance to SO_2_ and β-glucosidase activity (i.e., high, intermediate and low activity), were selected for whole genome sequencing and subjected to further characterization.

### Genome Sequencing, Assembly and Functional Annotation of Selected *H. uvarum* Strains

Summary statistics of the genome assemblies of the H2, H4, and H20 strains are presented in Table 3. The complete genome sequences of H2, H4, and H20 *H. uvarum* strains have been deposited in NCBI and are available from Genbank server under the following accessions numbers: uvarum H2 SAMN12284349; uvarum H4 SAMN12284353; uvarum H20 SAMN12284351.

**TABLE 3:** Summary of genome assembly for H2, H4, and H20 *H. uvarum* strains.

	*H. uvarum*	*H. uvarum*	*H. uvarum*
	strain H2	strain H4	strain H20
Genome size (Mb)	8,99	9,36	8,93
GC content (%)	31,09	31,41	31,43
N. of contigs	416	2368	296
N. of scaffolds	340	1346	180
N50 (Kb)	307	197	412
Genes	4128	4149	4137
Unique Genes	36	13	8

All the assemblies are similar in size -ranging from 8,93 Mb in H20 to 9,36 Mb in H4- and composition, with a GC content of approximately 31%. While the H2 and H20 assemblies show a significantly reduced number of contigs (416 and 296 for H2 and H20 respectively, versus 2368 for H4) and an increased overall contiguity (N50 307 and 412 Kb for H2 and H20, 197 Kb for H4) if compared with the assembly obtained for the H4 isolate. This observation is likely a reflection of an overall increase in the number and extent of repetitive sequences in the genome of H4.

Notwithstanding the difference in the contiguity of the assemblies equivalent numbers of genes (about 4000) were predicted for all the assemblies by the Augustus program.

Genome assemblies of *H. uvarum* H2, H4, and H20 were compared with a selection of the currently publicly available *Hanseniaspora* genomes, including five strains of *H. uvarum* and other 10 species of *Hanseniaspora* (Table 4).

**TABLE 4:** Summary of comparative genomic analysis of *Hanseniaspora* and *H. uvarum* strains.

			Genome	GC
		Unique	size	content
Hanseniaspora species	Genes	genes	(Mb)	(%)
*Hanseniaspora (spHNB-2018a)*	5146	635	10,31	36,47
*H. guilliermondii, (strain UTAD222)*	4104	56	9,15	30,56
*H. osmophila (strain AWRI3579)*	4694	138	11,60	36,24
*H. valbyensis (strain NRRL Y-1626)*	4475	898	11,61	31,17
*H. opuntiae (strain AWRI3578)*	4193	100	8,94	34,25
*H. pseudoguilliermondii (strain ZIM213)*	4235	114	5,94	34,04
*H. clermontiae (strain NRRL Y-27515)*	4305	221	5,69	36,29
*H. singularis (strain ZIM2326)*	4031	284	6,79	25,95
*H. vinae (strain T02/05AF)*	4823	121	7,30	37,00
*H. vinae (strain T02/19AF)*	4743	113	11,53	22,29
*H. uvarum (strain AWRI3580)*	4095	49	8,92	31,20
*H. uvarum (strain 34-9)*	3995	75	8,20	31,56
*H. uvarum (strain DSM2768)*	4379	393	9,62	32,17
*H. uvarum (strain CBA6001)*	4855	556	9,08	32,30
*H. uvarum (strain AWRI3581)*	4125	42	8,94	31,27
*H. uvarum (strain H2)*	4128	36	8,99	31,09
*H. uvarum (strain H4)*	4149	13	9,36	31,41
*H. uvarum (strain H20)*	4137	8	8,93	31,43

**FIGURE 2:**
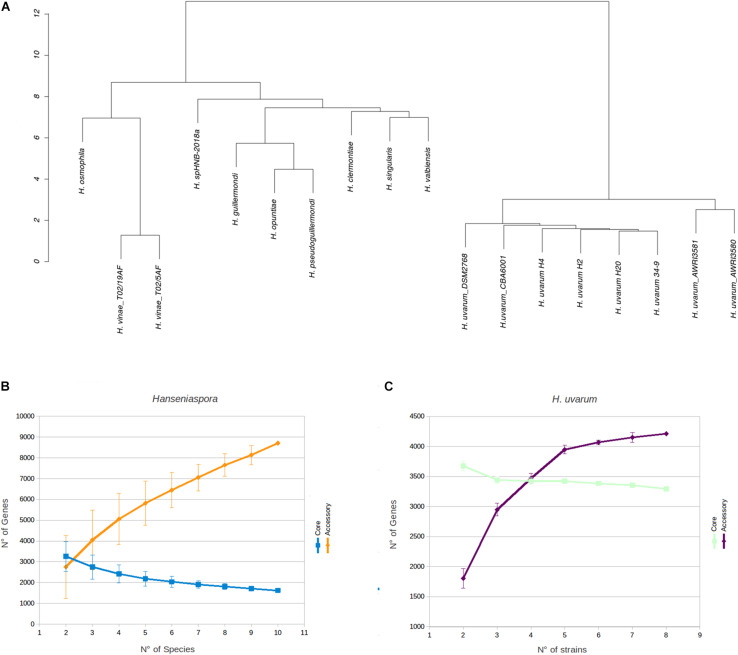
Phenetic clustering of *Hanseniaspora* and rarefaction analyses. **(A)** Hierarchical clustering of *Hanseniaspora* based on genomic identity. Pairwise levels of genomic identity were established by pairwise genome alignment, after masking dimorphic sites in each genome. Clustering was performed based on Euclidean distances of identity profiles. **(B)** Estimation of completeness of core and accessory genomes by rarefaction analyses at the genus level. Core and accessory genome sizes were calculated for all possible combinations of *Hanseniaspora* genomes. One representative species was selected for each genus included in the analyses (see section Materials and Methods). **(C)** Estimation of completeness of core and accessory genomes by rarefaction analyses for *H. uvarum*. Core and accessory genome sizes were calculated on each possible randomly resampled combinations of *H. uvarum* genomes.

Phenetic clustering of *Hanseniaspora* based on genome identity levels (Figure 2A, see section Materials and Methods) suggest that, notwithstanding slightly different levels of heterozygosity (Supplementary Table S1), all the *Hanseniaspora uvarum* strains included in our analyses form a well supported monophyletic clade, with an average level of genomic identity of 96.75% or higher. Importantly, while the clusters identified by our analyses are broadly consistent with the phylogeny of *Hanseniaspora* reported by Steenwyk et al. (2019) and correctly separate fast and slow evolving lineages, we notice some inconsistencies with respect to the position of *H. clermontiae*, which in our clustering seems to be closely related to the *H. singularis* and *H. valbiensis*. Considerations regarding cross-species average genomic identity levels of *Hanseniaspora* which are remarkably similar (all between 74 and 77% with an average of 76.45%) suggest that this observation is likely the reflection of the reduced resolution of simple methods based on average genomic identity, if compared with sophisticated methods based on complex evolutionary models, in the precise reconstruction of phylogenetic relationships between distantly related species.

To avoid possible ascertainment biases all the genomes were subjected to re-annotation using the Augustus program. Predicted genes were subjected to functional annotation of protein domains by the means of the pfam_scan software (see Section Materials and Methods).

Pairwise comparisons of sequence similarity of the predicted proteomes were performed using the blastP program, with the BLOSUM80 similarity matrix. Putative Clusters of Orthologous Genes (COGs) were subsequently established as groups of best reciprocal Blast hits, using a custom Perl script (see Section Materials and Methods). For each assembly, genes that were not assigned to COGs (i.e., did not recover a possible ortholog in any of the genomes included in the analysis) were considered “unique,” that is specific to a particular *Hanseniaspora* strain or species. Our approach identified a total of 7503 clusters (more than one gene) of putative orthologs as well as 3852 unique genes.

Summary statistics of gene content and numbers of “unique” genes are reported in Table 4.

The complete annotation of PFAM protein domains identified in all the “species-specific” genes is available at the following publicly accessible repository: https://github.com/matteo14c/supplementary_dataset_Guaragnella_et_al. Some of them have been selected for their biotechnological potential and reported on a heatmap (Supplementary Table S5 and Supplementary Figure S1).

Unsurprisingly -in the light of the relatively high levels of similarity between the genomes – we notice that all the *H. uvarum* strains herein considered show a very limited number of “unique genes” (between 126 for CBA6001 and 5 AWRI3580 and AWRI3581). While the number of unique genes associated with different species of *Hanseniaspora* is substantially larger (between 100 *H. opuntiae* and 898 *H. valbyensis*). This observation is also confirmed by rarefaction analyses. Indeed while estimates of the size of the core genome (Figure 2B) remain fairly stable when additional strains are sampled, suggesting that the currently available genomes provide an almost complete representation of the catalog of core genes *Hanseniaspora*, the accessory genome remains rather open when additional species are included (Figure 2B), indicating that our sampling of the *Hanseniaspora* species complex is probably incomplete.

**FIGURE 3:**
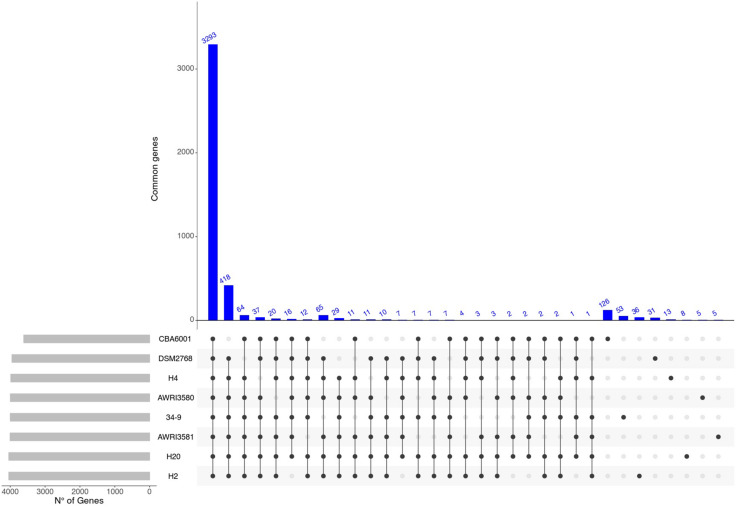
Graphical overview of shared genes. Number of genes shared between different combinations of *H. uvarum* strains. Blue bars indicate the number of shared genes. Gray bars are used to show the total number of genes for each *H. uvarum* strains.

On the other hand (Figures 2C, 3) profiles of gene absence/presence between the 8 *H. uvarum* strains included in this study (Figure 3) are completely consistent with a compact and relatively closed pan genome, as outlined by the fact that being composed by more than 3200 genes, the core genome accounts for more than 75% of the average (4223 genes) gene content of an isolate. Consistent with this observation, our analyses suggest that the 8 genomes included in this study provide a quite complete representation of the pan genome of *H. uvarum*, which seems to be composed by less than 4500 genes (Figure 2C).

In order to identify possible metabolic pathways or phenotypic traits specific for the H2, H20, and H4 *H. uvarum* strains, functional enrichment analyses of PFAM domains were performed and occurrences of protein domains in each isolate were compared with the equivalent figure in the *H. uvarum* pan genome by the means of a statistical test based on the hypergeometric distribution (see Section Materials and Methods).

The complete results of this analysis are reported in Supplementary Tables S2–S4. The 10 most over and under-represented domains for each strain are reported in Tables 5–7 for H2, H4, and H20 respectively. Interestingly, contrasting patterns of enrichment for selected domains were observed between the H2, H4 and H20 strains. In this regards, while H2 shows a highly significant enrichment of terms related to flocculin repeat (PF00624) and flocculation (Flo11 (PF10182) (Table 5), flocculation related protein domains such as PA14_2 (PF10528) are consistently under-represented in H4 (*p*-value 0.024) (Table 6). While we cannot conclusively exclude the possibility that under-representation of Flocculin domains in the H4 genome could be associated with the reduced contiguity of underlying genome assembly resulting in a reduced representation of repetitive of genes associated with repetitive sequence domains in the assembly, considerations regarding the average levels of aminoacidic identity of Flocculin genes (82.5%) and Flocculin domains (92.04%) advocates against this possibility. Indeed, such high levels of diversity are not likely to have a major impact on the assembly. Moreover, analysis based on coverage levels of the contigs (data not shown) suggests that all the Flocculin genes identified in the present study are present in single copy. No significantly over or under-representation of flocculation related terms (PA14_2, *p*-value 0.254) are observed for H20 (Table 7). A significant enrichment in the OPT domain (PF03169, *p*-value < 0.05) found in oligopeptide transporters was observed in both H2 and H4 strains. H4 strain showed also the presence of a PTR2 (PF00854) domain encoding for an oligopeptide transporter (*p*-value < 0.062). In order to verify whether this functional prediction was confirmed at lab-scale, flocculation tests were performed on the three strains. Results obtained confirm at least in part the observations based on genetic data: indeed while a strong flocculation phenotype (value 5) is observed in H2, on the other hand, H4 shows reduced levels of flocculation (value 2) and in H20 no flocculation could be observed (value 0).

**TABLE 5:** The top 10 enriched and under-represented domains of H2 strain.

Annotation	Gene name	Functional category	Occurrence	^∗^Tot	^∗∗^Occurrence bg	^∗∗∗^Tot bg	p_over
PF01735	PLA2_B	Lipid metabolism	7	5839	16	30370	0.005
PF00624	Flocculin	Flocculation	4	5839	163	30370	0.006
PF00244	14-3-3 protein	Cell signaling	2	5839	4	30370	0.024
PF03169	OPT	Oligopeptide transport	16	5839	55	30370	0.026
PF10182	Flo 11	Flocculation	1	5839	2	30370	0.037
PF10215	Ost4	Protein modification	1	5839	2	30370	0.037
PF01269	Fibrillarin	RNA processing	2	5839	5	30370	0.052
PF11838	ERAP1_C	Proteolysis	5	5839	16	30370	0.069
PF00119	ATP-synt_A	Oxidative phosphorylation	2	5839	6	30370	0.090
PF00115	COX1	Oxidative phosphorylation	2	5839	6	30370	0.090

**Annotation**	**Gene name**	**Functional category**	**Occurrence**	**^∗^Tot**	**^∗∗^Occurrence bg**	**^∗∗∗^Tot bg**	**p_under**

PF00399	PIR	Cell wall	2	5839	102	30370	0.000
PF13634	Nucleoporin_FG	Molecular transport	7	5839	77	30370	0.012
PF00078	RVT_1	Transcription	2	5839	27	30370	0.086
PF00364	Biotinjipoyl	Protein modification	7	5839	50	30370	0.229
PF02776	TPP_enzyme_N	Protein modification	4	5839	32	30370	0.236
PF00647	EF1G	Protein synthesis	1	5839	13	30370	0.255
PF13344	Hydrolase_6	Protein degradation	3	5839	25	30370	0.264
PF00406	ADK	Energy homeostasis	4	5839	30	30370	0.290
PF02775	TPP_enzyme_C	Protein	3	5839	23	30370	0.329
PF01423	LSM	m-RNA processing and regulation	12	5839	72	30370	0.354

*^∗^Tot, total numbers of domains; ^∗∗^ Occurrence bg, total occurrence in *H. uvarum* genomes; ^∗∗∗^ Tot bg, total number of domains in *H. uvarum*.*

**TABLE 6:** The top 10 enriched and under-represented domains of H4 strain.

Annotation	Gene name	Functional category	Occurrence	^∗^Tot	^∗∗^Occurrence bg	^∗∗∗^Tot bg	p_over
PF01179	Cu_amine_oxid	Aminoacids metabolism	3	5817	7	30370	0.029
PF10215	0st4	Protein modification	1	5817	2	30370	0.037
PF03169	OPT	Oligopeptide transport	15	5817	55	30370	0.049
PF07819	PGAP1	Protein transport	4	5817	12	30370	0.062
PF00854	PTR2	Oligopeptide transport	4	5817	12	30370	0.062
PF00119	ATP-synt_A	Oxy dative phosphorylation	2	5817	6	30370	0.089
PF00115	C0 × 1	Oxy dative phosphorylation	2	5817	6	30370	0.089
PF03184	DDE_1	DNA cleavage	2	5817	6	30370	0.089
PF01221	Dynein_light	Cell division	2	5817	6	30370	0.089
PF06414	Zeta_toxin	Protein modification	2	5817	6	30370	0.089

**Annotation**	**Gene name**	**Functional category**	**Occurrence**	**^∗^Tot**	**^∗∗^Occurrence bg**	**^∗∗∗^Tot bg**	**p_under**

PF10528	PA14_2	Flocculation	3	5817	43	30370	0.024
PF00724	Oxidored_FMN	Metabolism	6	5817	47	30370	0.178
PF08240	ADH_N	Ethanol Metabolism	7	5817	53	30370	0.178
PF00347	Ribosomal_L6	Protein synthesis	2	5817	21	30370	0.204
PF07727	RVT_2	Reverse transcription	0	5817	7	30370	0.226
PF02776	TPP_enzyme_N	Protein modification	4	5817	32	30370	0.239
PF00647	EF1G	Protein synthesis	1	5817	13	30370	0.257
PF08567	TFIIH_BTFja62 N	DNA repair	0	5817	6	30370	0.279
PF03501	S10_plectin	RNA binding	0	5817	6	30370	0.279
PF00205	TPP_enzyme_M	Protein modification	2	5817	17	30370	0.341

*^∗^ Tot, total numbers of domains; ^∗∗^ Occurrence bg, total occurrence in *H. uvarum* genomes; ^∗∗∗^ Tot bg, total number of domains in *H. uvarum*.*

**TABLE 7:** The top 10 enriched and under-represented domains of H20 strain.

Annotation	Gene name	Functional category	Occurrence	^∗^Tot	^∗∗^Occurrence bg	^∗∗∗^Tot bg	p_over
PF00119	ATP-synt_A	Oxidative phosphorylation	2	5840	6	30370	0.090
PF00115	C0 × 1	Oxidative phosphorylation	2	5840	6	30370	0.090
PF01221	Dynein_light	Cell division	2	5840	6	30370	0.090
PF13418	Kelch_4	Cell polarity and morphology	2	5840	6	30370	0.090
PF03501	S10_plectin	RNA binding	2	5840	6	30370	0.090
PF08567	TFIIH_BTF_p62_N	DNA repair		5840	6	30370	0.090
PF06414	Zeta_toxin	Protein modification	2	5840	6	30370	0.090
PF12348	CLASP_N	Cell division and dynamics	1	5840	3	30370	0.097
PF10513	EPL1	Transcription	1	5840	3	30370	0.097
	F-box	Protein degradation	1	5840	3	30370	0.097

**Annotation**	**Gene name**	**Functional category**	**Occurrence**	**^∗^Tot**	**^∗∗^Occurrence bg**	**^∗∗∗^Tot bg**	**p_under**

PF00078	RVT_1	Transcription	3	5840	27	30370	0.209
PF07727	RVT_2	Reverse transcription	0	5840	7	30370	0.224
PF02776	TPP_enzyme_N	Protein modification	4	5840	32	30370	0.236
PF10528	PA14_2	Flocculation	6	5840	43	30370	0.254
PF00347	Ribosomal_L6	Protein synthesis	3	5840	21	30370	0.404
PF00098	zf-CCHC	Transcription	12	5840	69	30370	0.419
PF08242	Methyltransf_12	Protein modification	0	5840	4	30370	0.426
PF00244	14-3-3 protein	Cell signaling	0	5840	4	30370	0.426
PF00724	Oxidored_FMN	Metabolism	8	5840	47	30370	0.436
PF04757	Pex2_Pex12	Peroxisome biogenesis	1	5840	9	30370	0.460

*^∗^ Tot, total numbers of domains; ^∗∗^ Occurrence bg, total occurrence in *H. uvarum* genomes; ^∗∗∗^ Tot bg, total number of domains in *H. uvarum*.*

## Discussion

Over the last years, the beneficial contribution of non-*Saccharomyces cerevisiae* yeast species to wine characteristics has been recognized, making the exploitation of non-conventional yeasts as a new source of biodiversity with potential biotechnological significance (Masneuf-Pomarede et al., 2015). Among these yeasts, the genus *Hanseniaspora*, which can play a critical role in the modulation of the wine sensory profile by increasing its complexity and organoleptic richness, is attracting a significant interest (Fleet, 2003). So far, the knowledge on genetics and physiology of *Hanseniaspora* species remains limited, notwithstanding some recent significant studies open new perspectives in the field, revealing species-specific properties to be explored (Langenberg et al., 2017; Seixas et al., 2019). In this context, genomics analysis may enable a correlation between genetics and useful traits, which could provide a roadmap for biotechnological exploitations (Hittinger et al., 2015; Riley et al., 2016).

Here we present *de novo* genome sequencing of three *Hanseniaspora uvarum* indigenous wine strains and comparative genomic analyses of *Hanseniaspora* at species and genus level. Among 26 isolates from various geographical locations or sources (Table 1), three of them, H2, H4, and H20, isolated from spontaneous grape must fermentation were selected and subjected to further characterization. H2, H4, and H20 showed heterogeneity for relevant genotypic and phenotypic features of oenological interest, such as β-glucosidase activity and the resistance to SO_2_ under fermentative conditions at laboratory scale (Figure 1 and Table 2) (Fleet and Heard, 1993; Rodriguez et al., 2004; Jolly et al., 2014).

Whole-genome sequencing revealed comparable genome size (∼9 Mb), GC content (∼31%) and number of genes (∼4000) for the three strains (Table 3). These data also converge on the genomic features of the other *H. uvarum* strains analyzed in this work (Table 4) and by other authors (Langenberg et al., 2017). Hierarchical clustering of *Hanseniaspora* species indicates that the *H. uvarum* strains included in this study form a well-supported monophyletic clade (Figure 2A), with high levels of genomic identity and a relatively compact pan-genome, where core genes account for more than 75% of the average number of genes that are annotated in any individual strain (Figure 2C). Unsurprisingly these considerations can be extended also to all members of the genus *Hanseniaspora*, which display relatively compact genomes containing a moderate number of genes. Consistent with these observations we notice that core genes constitute a consistent proportion (between 28 and 36%) of the average gene content of any *Hanseniaspora* species (Figure 2B), suggesting that the currently available data provide an almost complete representation of the core- genome of *Hanseniaspora*. This notwithstanding our results indicate also that accessory genome of *Hanseniaspora* remains relatively open, and that all in all currently available data offer only a partial representation of the pan-genome of *Hanseniaspora*.

On the other hand, intra-species analyses of patterns of functional annotation show distinct patterns of enrichment for several PFAM protein domains in the three strains. In particular, the significant difference with respect to terms related to flocculin repeats, found in lectin-like proteins, and flocculation (Flo11) observed in H2 and H4 strains is an intriguing and industrially relevant trait (Tables 5, 6). Flocculation, the process by which yeast cells spontaneously aggregate to form flocs with sediment in the culture, has been observed in different yeast species, including non-*Saccharomyces* isolates and *H. uvarum* (Rossouw et al., 2015). Beyond its physiological relevance as a protective mechanism to enhance the survival under environmental stresses (Marika et al., 1993), flocculation is a desirable technological feature allowing the separation of cells from media in fermentation processes such as brewing and winemaking (Pretorius, 2000; Soares, 2011), in particular sparkling wine obtained by the so-called Method Champenoise. This characteristic allows the rapid clarification and reduction of the handling of final products, with a significant decrease in production costs (Tofalo et al., 2016b; Vigentini et al., 2017). Moreover, yeast flocculation seems to be associated with the enhancement of ester production (Pretorius, 2000). Although the interest in non- *Saccharomyces* yeasts for use in sparkling wine production has increased only in recent years, different studies demonstrated that these yeasts can influence the aromas of sparkling wines through production of enzymes and metabolites during aging in contact with yeast lees (Ivit and Kemp, 2018). It is of note that lab-scale tests performed in this work on the three strains showed a degree of flocculation which mirrors the genetic prediction with a strong flocculation phenotype for H2 and a gradual decrease in the capacity to flocculate for H4 and H20 (data not shown). This confirms that flocculation capacity, as other physiological properties of oenological interest, is strain-dependent in *H. uvarum* (Romano et al., 1992, 1997; Ciani and Maccarelli, 1998; Caridi and Ramondino, 1999). In this context, the strain H2 characterized by high flocculation ability and high β-glucosidase activity might be considered as a suitable candidate for the production of traditional method sparkling wine with specific sensory attributes and distinctive characters. Additionally, its high resistance to SO_2_ might favor the persistence of this non-*Saccharomyces* strain during fermentation as reported in Grangeteau et al. (2016).

Another genetic trait of biotechnological significance is the enrichment in domains involved in oligopeptides transport: the OPT domain (PF03169) shared by H2 and H4 strains and the PTR2 domain (PF00854) found only in H4 (Tables 5, 6). In general, oligopeptides transport affects both fermentation and the formation of wine aroma by mediating nitrogen utilization, storage and mobilization in yeasts. Particularly, it has been demonstrated that the performance and fitness of *S. cerevisiae* cells using a higher amount of oligopeptides from grape must is due to metabolic effects (Marsit et al., 2016). The relevance of metabolic control in the organoleptic profile of the wine could be also related to the over-represented domains PLA2_B (PF01735) and Cu_amine_oxidase (PF01179), found in H2 and H4 strains and involved in lipid and aminoacids metabolism, respectively (Tables 5, 6). These identified genetic features reveal a promising potential of these indigenous strains to be applied in fermentation processes and modulation of wine flavor and aroma.

The over-representation of the two domains COX1 (PF00115) and ATP-synt_A (PF00119) encoding for putative proteins involved in oxidative phosphorylation clearly indicates conserved regions deriving from the mitochondrial genome (Tables 5–7). Since the mitochondrial genome of *H. uvarum* exhibits unique features in terms of organization and molecular architect, this point could become an object for further investigation (Pramateftaki et al., 2006).

Overall these data contribute to increase the catalog of publicly available genomes from *H. uvarum* strains isolated from natural grape samples and provide a good starting point for unraveling the biodiversity and the biotechnological potential of this non-*Saccharomyces* yeast species at the genus, species and strain levels in oenological applications (Martin et al., 2018).

## Data Availability Statement

Whole Genome Assemblies of *Hanseniaspora uvarum* strains H4, H20, and H2 have been deposited at GenBank under the following accession numbers: WEHR00000000, WEHS00000000 and WEHT00000000. Complete annotations of PFAM domains and inferred clusters of orthologous genes are available at: https://github.com/matteo14c/supplementary_dataset_Guaragnella_et_al.

## Author Contributions

All authors significantly contributed to this manuscript. RP, GS, and CM performed the experiments. MC performed the bioinformatics analyses. NG, MC, and AC performed the data curation. NG, AC, PR, and GP designed and supervised the different parts of the study. NG, MC, and AC wrote the first draft of the manuscript. All authors contributed to revisions of the manuscript and approved the final version. GP, AC, and PR contributed to the funding acquisition.

## Conflict of Interest

The authors declare that the research was conducted in the absence of any commercial or financial relationships that could be construed as a potential conflict of interest.

## Supplementary Material

FIGURE S1 Heatmap of the number of species-specific genes associated with selected PFAM domains. The heatmap displays the number of species-specific genes associated with biotechnologically relevant PFAM domains. Dark blue indicates high values. Light blue low values. Unity based normalization is applied to the columns of the heatmap (i.e., the domains) to facilitate the comparison.

TABLE S1 Annotation of heterozygous sites and availability of raw sequencing data. Full names of all the *Hanseniaspora* species included in this study are reported in the first column. In the second column, “yes” indicates that annotation of heterozygous sites is provided in the reference assembly, a “no” indicates that this information is not available. Availability of raw sequencing data (yes available, no not available) are reported in the 3rd column.

TABLES S2–S4 Over and under representation of protein PFAM domains in the 3 *H. uvarum* genomes assembled in this study. Names of annotated PFAM domains are reported in the first column. The 2nd and 3rd column report, respectively the total number of occurrences of each domain, and the total number of annotated PFAM domains identified for that species. The 4th and the 5th column contain the equivalent figures (occurrences each domain and total number of domains annotated) for the *H. uvarum* pan-genome. *p*-values for under and over-representation are reported in the 6th and 7th column respectively.

TABLE S5 Occurrence of selected PFAM domains in species-specific genes. Domains are indicated in the rows and species in the columns.
