# Corrigendum: Genome Sequencing and Comparative Analysis of Three *Hanseniaspora uvarum* Indigenous Wine Strains Reveal Remarkable Biotechnological Potential

**DOI:** 10.3389/fmicb.2020.01169

**Published:** 2020-06-09

**Authors:** Nicoletta Guaragnella, Matteo Chiara, Angela Capece, Patrizia Romano, Rocchina Pietrafesa, Gabriella Siesto, Caterina Manzari, Graziano Pesole

**Affiliations:** ^1^Institute of Biomembranes, Bioenergetics and Molecular Biotechnologies, CNR, Bari, Italy; ^2^Department of Biosciences, Biotechnology and Biopharmaceutics, University of Bari “A. Moro”, Bari, Italy; ^3^Dipartimento di Bioscienze, Università degli Studi di Milano, Milan, Italy; ^4^Scuola di Scienze Agrarie, Forestali, Alimentari ed Ambientali, University of Basilicata, Potenza, Italy

**Keywords:** non-*Saccharomyces* yeasts, *Hanseniaspora uvarum*, genome sequencing and annotation, *Hanseniaspora* species, comparative genomics, flocculation

In the original article, there was an error as the extent of knowledge on genetics and physiology of the *Hanseniaspora* species was not correctly identified.

A correction has been made to the **Discussion** section, paragraph 1. The corrected sentence appears below:

“Over the last years, the beneficial contribution of non-*Saccharomyces cerevisiae* yeast species to wine characteristics has been recognized, making the exploitation of non-conventional yeasts as a new source of biodiversity with potential biotechnological significance (Masneuf-Pomarede et al., 2015). Among these yeasts, the genus *Hanseniaspora*, which can play a critical role in the modulation of the wine sensory profile by increasing its complexity and organoleptic richness, is attracting a significant interest (Fleet, 2003). So far, the knowledge on genetics and physiology of *Hanseniaspora* species remains limited, notwithstanding some recent significant studies open new perspectives in the field, revealing species-specific properties to be explored (Langenberg et al., 2017; Seixas et al., 2019). In this context, genomics analysis may enable a correlation between genetics and useful traits, which could provide a roadmap for biotechnological exploitations (Hittinger et al., 2015; Riley et al., 2016).”

The authors apologize for this error and state that this does not change the scientific conclusions of the article in any way. The original article has been updated.
