# Supplementary figures and images for: Genome Sequencing and Comparative Analysis of Three Hanseniaspora uvarum Indigenous Wine Strains Reveal Remarkable Biotechnological Potential
Source: Front Microbiol. 2020 Jan 21;10:3133. doi: 10.3389/fmicb.2019.03133 (PMC6986195; doi:10.3389/fmicb.2019.03133)

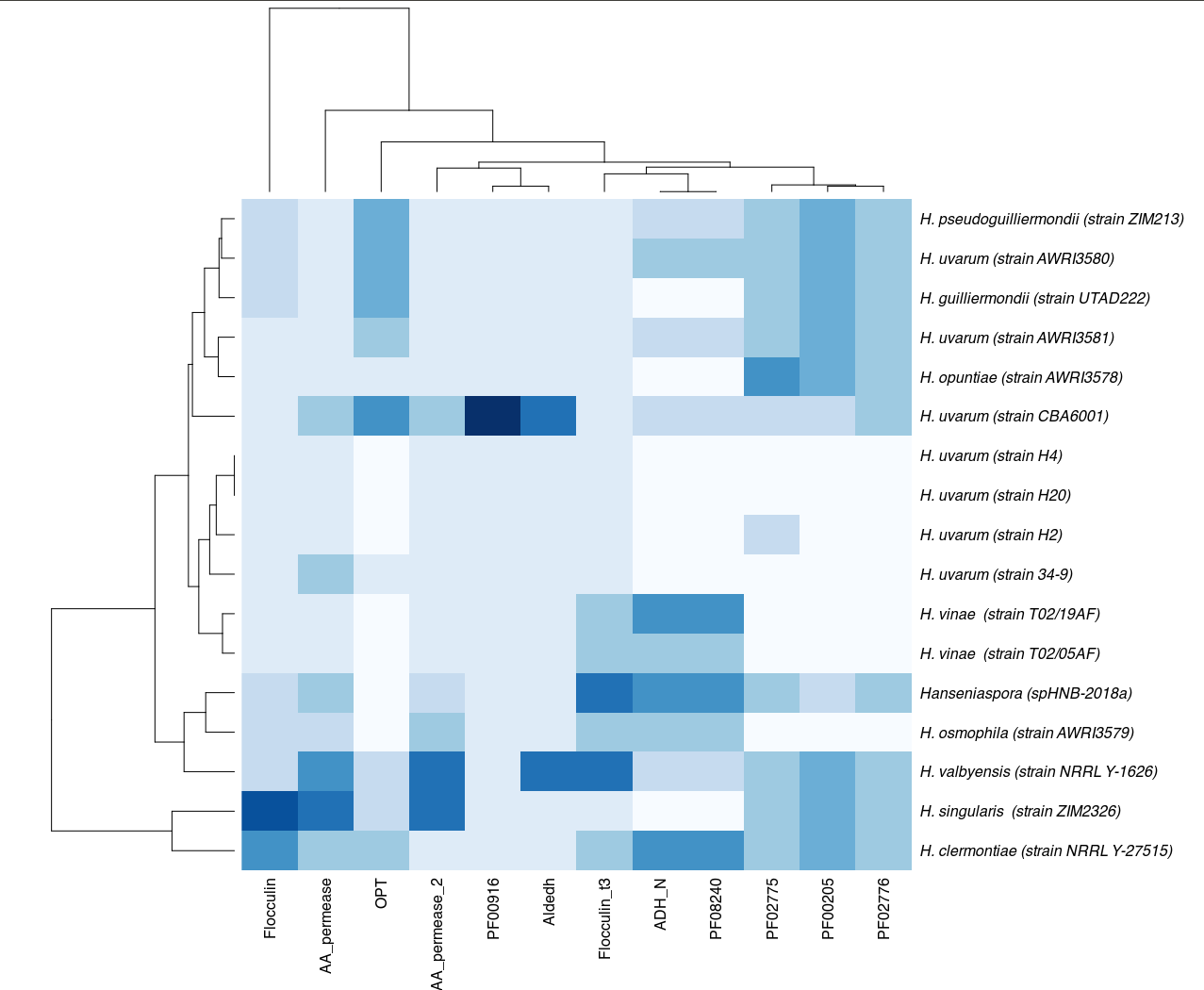

Supplement: FIGURE S1 — Heatmap of the number of species-specific genes associated with selected PFAM domains. The heatmap displays the number of species-specific genes associated with biotechnologically relevant PFAM domains. Dark blue indicates high values. Light blue low values. Unity based normalization is applied to the columns of the heatmap (i.e., the domains) to facilitate the comparison. [file Image_1.PNG]
